# Examining how changes in provincial policy on vape marketing impacted the distribution of vaping advertisements near secondary schools in London, Ontario

**DOI:** 10.17269/s41997-020-00453-9

**Published:** 2021-01-06

**Authors:** Gina Martin, Drew D. Bowman, Megan Graat, Andrew F. Clark, Alexander J. Wray, Zoe Askwith, Jamie A. Seabrook, Jason A. Gilliland

**Affiliations:** 1 Human Environments Analysis Laboratory, Western University, Room 2432, SSC, London, ON N6A 5C2 Canada; 2 Children’s Health Research Institute, London, ON N6C 2V5 Canada; 3 Department of Geography and Environment, Western University, London, ON N6A 5C2 Canada; 4 School of Food and Nutritional Sciences, Brescia University College at Western University, London, ON N6G 1H2 Canada; 5 Lawson Health Research Institute, London, ON N6A 4V2 Canada; 6 Department of Paediatrics, Western University, London, ON N6A 5C1 Canada; 7 Department of Epidemiology & Biostatistics, Western University, London, ON N6A 5C1 Canada; 8 School of Health Studies, Western University, London, ON N6A 3K7 Canada

**Keywords:** Vaping, E-cigarettes, Marketing, Policy, Youth, Schools, Vapotage, cigarettes électroniques, marketing, politique (principe), adolescent, établissement scolaire

## Abstract

**Objectives:**

On January 1, 2020, the Government of Ontario passed a regulation banning vaping advertisements by retailers, apart from specialty shops. A motivation for this ban was to limit youth exposure to vaping advertisements. The primary goal of this research was to evaluate the impact of this ban on the number and density of vaping advertisements surrounding secondary schools. Additionally, we examined whether the number of vaping advertisements varied by school socio-demographic characteristics.

**Methods:**

This study used a pre-post design. Audits were conducted December 2019 (pre-ban) and again January to February 2020 (post-ban), to identify vaping advertisements within 800 m surrounding secondary schools (*n* = 18) in London, Ontario.

**Results:**

Prior to the ban, there were 266 vaping advertisements within 800 m of secondary schools. After the ban, this was reduced to 58, a 78.2% reduction. The mean number of vaping advertisements surrounding schools significantly decreased from 18.1 before the ban to 3.6 after the ban (*p* < 0.001). A significant positive correlation was found, prior to the ban, between the number of vaping advertisements surrounding schools and school-level residential instability (*r* = 0.42, *p* = 0.02). After the ban, no significant correlations were found between the number of vaping advertisements and school socio-demographic characteristics.

**Conclusion:**

The provincial ban of vaping advertisements in select retail settings significantly reduced the number of vaping advertisements in the areas surrounding secondary schools in London, Ontario. The ban also reduced socio-demographic inequities in youths’ potential exposure to marketing of vaping products. Continued monitoring of the geographic accessibility and promotion of vaping products is warranted.

## Résumé

**Objectifs:**

Le 1^er^ janvier 2020, le gouvernement de l’Ontario adoptait un règlement interdisant les annonces publicitaires sur le vapotage dans les magasins de détail, sauf dans les boutiques spécialisées. L’une des raisons de cette interdiction était de limiter l’exposition des jeunes à la publicité sur le vapotage. Notre étude visait principalement à évaluer les incidences de l’interdiction sur le nombre et la densité des annonces publicitaires sur le vapotage autour des écoles secondaires. Nous avons aussi cherché à déterminer si le nombre d’annonces publicitaires sur le vapotage variait selon le profil sociodémographique des écoles.

**Méthode:**

Un protocole avant-après été utilisé pour cette étude. Des audits ont été menés en décembre 2019 (avant l’interdiction), puis de nouveau en janvier et février 2020 (après l’interdiction) pour dénombrer les annonces publicitaires sur le vapotage dans un rayon de 800 m des écoles secondaires (*n* = 18) de London, en Ontario.

**Résultats:**

Avant l’interdiction, il y avait 266 annonces publicitaires sur le vapotage dans un rayon de 800 m des écoles secondaires. Après l’interdiction, ce nombre n’était plus que de 58, soit une baisse de 78,2 %. Le nombre moyen d’annonces publicitaires sur le vapotage autour des écoles a diminué de façon significative, passant de 18,1 avant l’interdiction à 3,6 après (*p* < 0,001). Une corrélation positive significative a été observée, avant l’interdiction, entre le nombre d’annonces publicitaires sur le vapotage autour des écoles et l’instabilité résidentielle au niveau des écoles (*r* = 0,42, *p* = 0,02). Après l’interdiction, aucune corrélation significative n’a été observée entre le nombre d’annonces publicitaires sur le vapotage et le profil sociodémographique des écoles.

**Conclusions:**

L’interdiction provinciale des annonces publicitaires sur le vapotage dans certains magasins de détail a considérablement réduit le nombre de ces annonces dans les environs des écoles secondaires de London, en Ontario. L’interdiction a aussi réduit les iniquités sociodémographiques de l’exposition potentielle des jeunes au marketing des produits de vapotage. Il est justifié d’assurer une surveillance continue de l’accessibilité géographique et de la promotion des produits de vapotage.

## Introduction

Vaping among Canadian children and youth has increased substantially in recent years (Boak et al. 2020; Cole et al. 2020). Vaporizers or vapes (also known as electronic cigarettes, e-cigarettes, electronic nicotine delivery systems, vape mods, or vape pens) are devices that heat a liquid solution—commonly infused with nicotine, cannabis, and/or flavouring compounds—into an aerosol that is inhaled into the lungs (National Academies of Sciences and Engineering 2018). When nicotine vapes were first introduced to the global market, they were promoted for tobacco smoking cessation as a safer alternative to combustible tobacco. However, vaping has gained popularity among youth, including those who have never smoked tobacco (Cole et al. 2020). In 2019, 23% of Ontario students in grades 7–12 had used a vaping device in the past year and 13% reported use weekly or daily (Boak et al. 2020). Additionally, the majority of Canadian youth who vaped did so with an e-liquid containing nicotine (Statistics Canada 2020). As vaping may lead to nicotine dependence and the long-term impacts of use on children and youth are unknown (Miyashita and Foley 2020), its increasing prevalence among this age group is a critical public health concern.

As vaping among youth has increased, so have marketing expenditures (Azagba and Manzione 2020; Mantey et al. 2016). This is concerning, as youth are highly susceptible to advertisements for potentially harmful products, including vapes (Krugman 2016). An increasing body of literature recognizes the influence of advertising in the retail environment on health-related behaviours (Bowman et al. 2019; Mantey et al. 2016). For example, a study of students in New Jersey found that individuals attending schools with greater retailer-based vaping advertisements within a half-mile were significantly more likely to have vaped in the past month (Giovenco et al. 2016a). Moreover, youth report retailers (e.g., convenience stores, gas stations, and vape shops) as the most common locations for vaping advertisement exposure, with higher exposure reported in countries with less restrictive regulatory environments (Cho et al. 2019).

In the contemporary Canadian context, little is known regarding vape retailers in areas youth may frequent, such as near their school. One study found that the majority of Canadian secondary schools, in four provinces, did not have a specialty vape shop nearby in 2017; these data did not include gas stations or convenience stores (Cole et al. 2019), which represent a potential source of vaping advertisement exposure. A 2014 audit of retailers (including vape shops, tobacco shops, and convenience stores), in four Canadian cities, found that exterior vaping advertisements were rare (Hammond et al. 2015). However, in May 2018, an amendment to the *Tobacco Act*, entitled the *Tobacco and Vaping Products Act*, enabled tobacco retailers (e.g., convenience stores and gas stations) to sell and *promote* vaping liquids and devices as nicotine products (Government of Canada 2020a). Thus, the potential for exposure to vape advertisements in a retail environment increased after May 2018. Following this, the Government of Ontario amended the *Smoke-Free Ontario Act* to ban vape advertising by retailers, apart from vape and cannabis specialty shops, as of January 1, 2020 (Ontario Ministry of Health 2019). A main motivation of the provincial policy change was to prevent youth from being exposed to vaping advertisements in retail settings. In Ontario, an individual must be aged 19 or older to be permitted to enter a vape specialty shop.

The goal of this research was to evaluate the impact of the provincial ban of vaping advertisements in retail settings (with the exception of specialty shops) on the potential exposure of youth to vaping promotions (number and density of advertisements) in the areas surrounding secondary schools in London, Ontario. To our knowledge, no study has examined vaping advertisements in retail settings near schools in Canada. Additionally, no study has evaluated the impact of vaping advertisement restrictions on the number and density of advertisements surrounding secondary schools. We also examined whether the number and density of vaping advertisements varied by *school* socio-demographic characteristics, both before and after the advertising ban, as studies suggest that vape retailer locations might be geographically inequitable in relation to socio-demographic characteristics (Dai and Hao 2017; Giovenco et al. 2016a, b; Robitaille et al. 2019).

## Methods

This study uses a pre-post design to evaluate the impact of vaping advertisement restrictions on the number of vaping advertisements surrounding secondary schools (*n* = 18) in London, Ontario. These eighteen schools represent all secondary schools in the two largest school boards in London (Thames Valley District School Board and London District Catholic School Board), for which students’ socio-demographic data were available.

### Vape advertisement audits

To evaluate changes in vape advertising near schools, audits were conducted from December 2 to December 18, 2019 (pre-ban) and again from January 16 to February 6, 2020 (post-ban) within the area surrounding each school. Areas surrounding schools were defined by an 800-m Euclidean buffer around each school’s property boundary, which represents approximately a ten-minute walk for youth (Martin et al. 2019). Prior to the start of the audits, a database of potential vaping advertisement locations was created by identifying four types of sites where vaping advertisements could be displayed: (1) specialty shops (this included vape shops as well as cannabis shops, as sellers of vape products and devices); (2) convenience stores; (3) gas stations; and (4) billboards and transit shelters. Convenience stores and gas stations were identified from the Middlesex London Health Unit’s Public Health Inspector Database. Billboards and transit shelters were identified from the City of London’s Billboards dataset, available through their Open Data Portal. Online searches (including Google and Yellow Pages) were used to identify specialty vape and cannabis shops within the city. These data were uploaded to Collector for ArcGIS (ESRI, Redlands, CA, USA). Researchers used Collector on their smartphones, as it provided a convenient method to capture images of all vaping advertisements in the field and to log the number of advertisements at each location. Because online sources, such as Google, may have missed some specialty shops, a full street audit was completed of all commercial land use areas within 800 m of the schools.

Audits were conducted by trained researchers in teams of two, who surveyed the area within 800 m around each of the 18 schools to identify vaping advertisements. Once at a potential vape advertising location, the external advertisements of the property and building were examined to identify vaping advertisements. Advertisements were counted if they contained images of vaping products (e.g., devices, e-juice, flavour pods, brand names) or vaping-related phrases or words (e.g., vape, vaping, vaporizer, electronic cigarettes, e-cigarettes). The storefront signs of specialty shops were included. “No vaping” signs were excluded. Specialty retailer storefront signs are a potential location for vape promotion not specifically addressed under the *Smoke-Free Ontario Act.* Vaping advertisements that appeared more than once at the same retailer were counted individually.

No vaping advertisements were found on billboards or transit shelters either before or after the ban. Therefore, these location types were not included in further analysis.

### Measures

#### Vaping advertisements

The primary outcomes for this study were the number and density (advertisements/km^2^) of vaping advertisements near each school (within 800 m), pre- and post-ban. A weighted count was also derived for each school, based on the proportion of retailers with vaping advertisements from the total number of retailers (gas stations, convenience stores, and specialty shops) surrounding each school, to account for the fact that not all of the specified retailers sell vaping products (Giovenco et al. 2016a).

Vaping advertisements were also measured at 400 m to examine whether effects differed when potential advertising exposure was measured closer to schools. All measures were calculated using ArcMap version 10.7 (ESRI, Redlands, CA, USA).

#### School socio-demographic characteristics

Socio-demographic characteristics for each of the 18 schools were calculated by taking the proportion of students in the school who resided in a dissemination area (DA) classified as the most deprived quintile based on the Canadian Index of Multiple Deprivation (CIMD) for Ontario (CIMD-Ontario data are from Statistics Canada) (i.e., *% of students per school who live in a deprived area*). DAs are the smallest administrative area for which socio-demographic data are available from the Canadian Census; they typically contain 400–700 people (Statistics Canada 2019). Data on students’ DA of residence was obtained from an anonymized school bus eligibility database, which included residential postal codes for every student by school (but did not include any other information). The postal codes were used to link students to their respective DAs.

The CIMD was based on the 2006 Canadian Marginalization Index (CAN-Marg) (Matheson et al. 2012). The CIMD-Ontario is an index derived for each DA, specifically for Ontario, from the 2016 Census and is comprised of four dimensions of deprivation: (1) residential instability; (2) economic dependency; (3) ethno-cultural composition; and (4) situational vulnerability. The dimension of residential instability takes into consideration the tendency of inhabitants of a neighbourhood to change over time and includes five indicators (i.e., proportion of: dwellings that are apartment buildings; dwellings that are rented; persons living alone; population who moved in last 5 years; population who are single, divorced, separated, or widowed). Economic dependency considers reliance on the workforce or a dependence on sources of income other than employment; this dimension also includes five indicators (i.e., proportion of population who are: aged 65 and older; aged 15 and older who are not participating in the labour force; aged 0–14 and 65 and older divided by population aged 15–64; unemployed; receiving government transfer payments). Ethno-cultural composition considers the community make-up of immigrant populations and those who self-identify as a visible minority and includes four indicators (i.e., proportion of population who: are foreign-born; self-identify as visible minority; have no knowledge of English or French; are recent immigrants). Situational vulnerability takes into account factors such as educational attainment, housing conditions, and Indigenous identity; this dimension includes three indicators (i.e., proportion of: dwellings needing major repair; population who identify as Indigenous; population who are aged 25–64 without a high school diploma). Factor analysis was used to derive the CIMD-Ontario. A set of initial variables was selected for the factor analysis as established by the CAN-Marg and through expert consultation. Different indicator variables loaded within the four dimensions of deprivation to create the final index; some indicators were not included because they were not significantly correlated with any factors. (See Statistics Canada (2019) for more details.)

#### Commercial land use

The percent of land classified as commercial within the 800-m buffer surrounding each school was derived from City of London land use data. Commercial land use was considered as a co-variate because of the possibility that associations between school socio-demographic characteristics and number or density of vaping advertisements could be a function of municipal land use zoning and general commercial activity in the areas surrounding schools.

### Statistical analysis

To examine changes in vape advertising surrounding secondary schools, paired *t* tests were conducted on the vaping advertisement measures for 800-m and 400-m buffers. Cohen’s *d* was used to determine the effect size for the paired *t* test, where 0.20 indicates a weak effect, 0.50 a moderate effect, and 0.80 a large effect (Cohen 1988). To address any non-normality in the change from pre- to post-ban, we also utilized Yuen’s robust paired test. Yuen’s method addresses issues that arise from violating the normality assumption (Fradette et al. 2003).

A series of Kendall’s tau (*r*_Τ_) tests were used to assess correlations between vaping advertisement measures and school socio-demographic characteristics, before and after the vaping advertisement ban. Kendall’s tau is an appropriate statistic for data with a small n (Field et al. 2012). As recommended by Cohen (1988), effect sizes for Kendall’s tau correlations were considered small, medium, and large at 0.10, 0.30, and 0.50, respectively. Partial correlations were also examined, adjusting for commercial land use density (Kim 2015), as commercial land use density may have a confounding effect on the relationship between the number of vaping advertisements surrounding schools and the deprivation dimensions.

A *p* value < 0.05 was considered statistically significant. All analyses were conducted in R version 3.5.2.

## Results

**Fig. 1:**
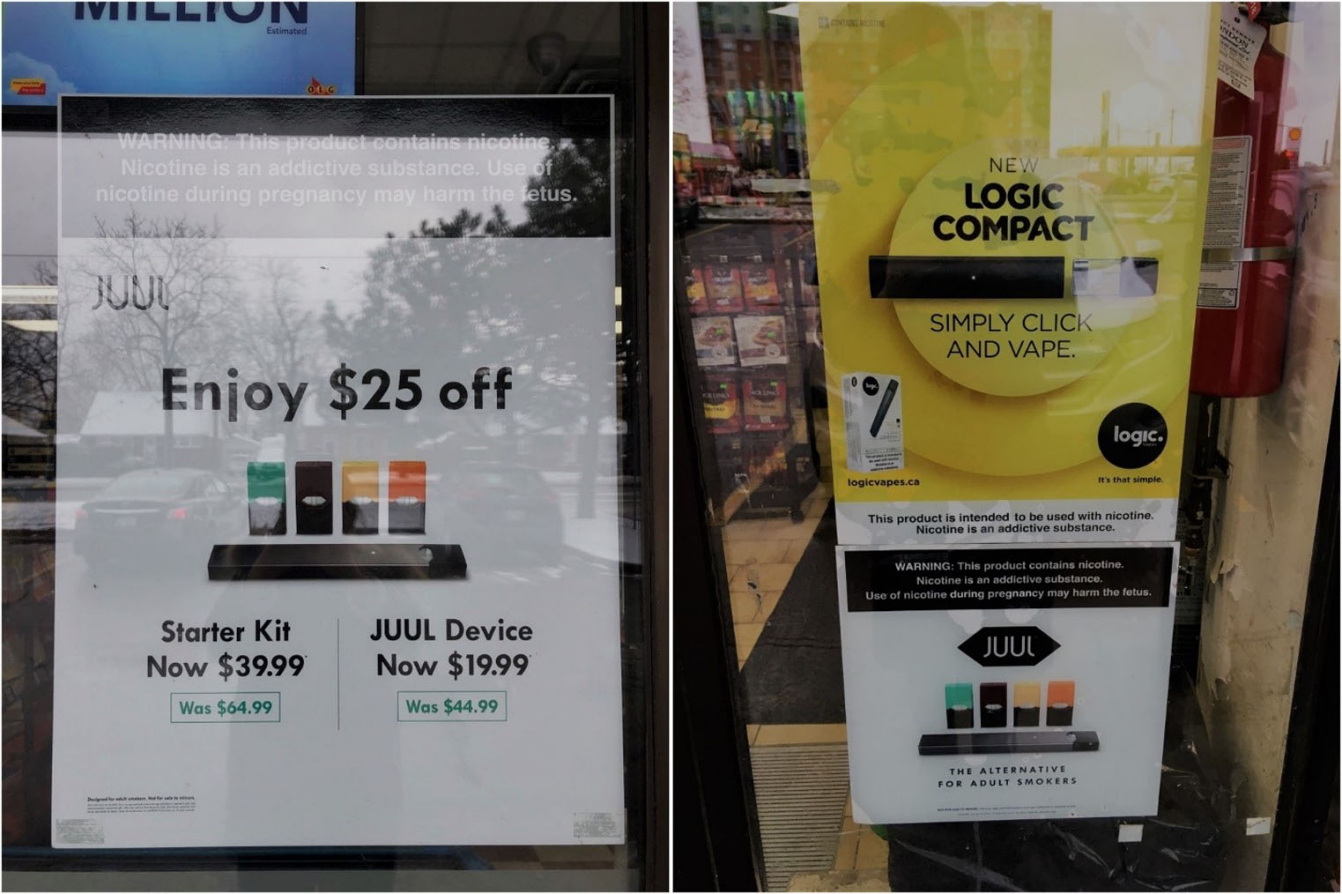
Exterior facing vaping advertisements at retailers from audits conducted December 2019

There were 100 retail sites identified within 800 m of the 18 secondary schools that could potentially advertise vape products (i.e., convenience stores, gas stations, and specialty shops). Of the 100 potential vaping retailers, 68 had at least one advertisement before the policy change, compared to only 16 thereafter (5 of which were gas stations or convenience stores, contravening the ban) (Table 1). Prior to the ban, there were 266 vaping advertisements within 800 m of secondary schools in London, Ontario. Figure 1 presents examples of vaping advertisements that were located near secondary schools prior to the ban. After the ban, this was reduced to 58 vaping advertisements, a reduction of 78.2% (six of these advertisements were from convenience stores or gas stations).

**Table 1:** Frequency and proportion of retailers with at least one vaping advertisement by all retailers who could potentially advertise vaping products pre- and post-ban, surrounding secondary schools

**400 m**	**Convenience stores (*n*** **= 19)**	**Gas stations (*n*** **= 6)**	Specialty stores^*^ **(*n*** **= 2)**	**Total (*n*** **= 27)**
Pre-ban	11 (58%)	4 (67%)	2 (100%)	17 (63%)
Post-ban	0 (0%)	1 (17%)	2 (100%)	3 (11%)
**800 m**	**Convenience stores (*n*** **= 66)**	**Gas stations (*n*** **= 23)**	Specialty stores^*^ **(*n*** **= 11)**	**Total (*n*** **= 100)**
Pre-ban	44 (67%)	13 (57%)	11 (100%)	68 (68%)
Post-ban	4 (6%)	1 (4%)	11 (100%)	16 (16%)

^*^Because storefront signs were included, all specialty shops had at least one advertisement

Fifteen schools (83.3%) had at least one vaping advertisement within 800 m prior to the advertising ban. Nine schools (50%) had at least one advertisement after the ban. The mean number of advertisements in the areas surrounding schools was 18.1 (minimum = 0, maximum = 74, SD = 20.7) before the ban; after the ban, the mean number decreased significantly to 3.6 (minimum = 0, maximum = 22, SD = 6.1) (*t* = 3.9, *p* < 0.001, *d* = 0.68). Yuen’s robust paired test also found a significant difference in pre- and post-ban mean number of vaping advertisements within 800 m of secondary schools (*Y* = 3.6, *p* < 0.01). The mean weighted number of advertisements also reduced significantly (*t* = 3.6, *p* < 0.01, *d* = 0.66; *Y* = 3.1, *p* < 0.01). On average, schools had 6.8 advertisements per km^2^, within 800 m, prior to the ban; this was reduced significantly to 1.4 after the ban (*t* = 3.6, *p* < 0.01, *d* = 0.65; *Y* = 3.2, *p* < 0.01) (Table 2).

**Table 2:** Vaping advertisements in the areas surrounding London, Ontario, secondary schools before and after the January 1, 2020 ban on vaping advertisements at convenience stores and gas stations, *n* = 18

	Mean (SD) pre-ban advertisements	Mean (SD) post-ban advertisements	Average change	*p*
Count—unweighted				
400 m	2.9 (3.8)	0.5 (1.2)	− 2.4	< 0.05
800 m	18.1 (20.7)	3.6 (6.1)	− 14.5	< 0.001
Count—weighted by the number of retailers with ads/total number of retailers				
400 m	1.9 (2.9)	0.1 (0.4)	− 1.8	< 0.05
800 m	12.2 (14.5)	0.8 (1.7)	− 11.4	< 0.01
Density—advertisements per km^2^				
400 m	3.3 (4.3)	0.6 (1.6)	− 2.7	< 0.05
800 m	6.8 (8.5)	1.4 (2.4)	− 5.5	< 0.01

*SD*, standard deviation

Based on analyses using a 400-m buffer, nine schools (50.0%) had at least one vaping advertisement prior to the ban; this was reduced to three schools (16.7%) after the ban. Of 27 potential vape advertising retailers within 400 m, 17 had at least one advertisement before the ban, compared to only 3 after. The mean number of advertisements within 400 m of schools was 2.9 (minimum = 0, maximum = 13, SD = 3.8) before the ban; after the ban, the mean number decreased significantly to 0.5 (minimum = 0, maximum = 4, SD = 1.3) (*t* = 2.7, *p* < 0.05, *d* = 0.55; *Y* = 2.5, *p* < 0.05). On average, schools had 3.3 advertisements per km^2^ prior to the ban; this was reduced significantly to 0.6 after (*t* = 2.8, *p* < 0.05, *d* = 0.56; *Y* = 2.7, *p* < 0.05) (Table 2).

There were no statistically significant correlations between vaping advertisements surrounding schools and school-level economic dependency or ethno-cultural composition, prior to the advertising ban. However, a significant positive correlation was found between vaping advertisement measures and school-level residential instability as well as situational vulnerability, in unadjusted correlations. After adjusting for commercial land use density, only residential instability was statistically significant; the effect size was medium. The change in vaping advertisements surrounding schools from pre- to post-ban was also positively related to school-level residential instability. After the advertising ban, there were no statistically significant correlations between vaping advertisements surrounding schools and any of the deprivation dimensions (see Table 3).

**Table 3:** Correlation between number of vape advertisements within 800 m of a secondary school and proportion of students per school residing in the most deprived quintile of the CIMD dimensions (*N* = 18), *r*_Τ_ (*p* value)

		Residential instability	Economic dependency	Ethno-cultural composition	Situational vulnerability
Unadjusted					
Pre-ban	Count (unweighted)	*0.51 (< 0.01)*	0.03 (0.88)	− 0.11 (0.54)	*0.36 (0.04)*
	Count (weighted)	*0.52 (< 0.01)*	0.04 (0.82)	− 0.11 (0.54)	*0.36 (0.04)*
	Density	*0.51 (< 0.01)*	0.01 (0.94)	− 0.13 (0.45)	*0.36 (0.04)*
Post-ban	Count (unweighted)	0.27 (0.15)	− 0.02 (0.90)	− 0.30 (0.11)	0.19 (0.31)
	Count (weighted)	0.24 (0.19)	− 0.06 (0.75)	− 0.30 (0.10)	0.17 (0.37)
	Density	0.26 (0.15)	− 0.05 (0.78)	− 0.32 (0.08)	0.17 (0.35)
Difference (counts pre- to post-ban)		*0.52 (< 0.01)*	0.01 (0.94)	− 0.09 (0.59)	*0.39 (0.03)*
Adjusted^*^					
Pre-ban	Count (unweighted)	*0.42 (0.02)*	− 0.13 (0.48)	− 0.07 (0.68)	0.31 (0.09)
	Count (weighted)	*0.41 (0.02)*	− 0.11 (0.54)	− 0.07 (0.68)	0.30 (0.09)
	Density	*0.41 (0.02)*	− 0.14 (0.42)	− 0.11 (0.55)	0.30 (0.09)
Post-ban	Count (unweighted)	0.12 (0.49)	− 0.14 (0.42)	− 0.30 (0.10)	0.11 (0.53)
	Count (weighted)	0.11 (0.52)	− 0.17 (0.33)	− 0.30 (0.10)	0.09 (0.62)
	Density	0.13 (0.48)	− 0.17 (0.33)	− 0.32 (0.07)	0.09 (0.60)
Difference (counts pre- to post-ban)		*0.42 (0.02)*	− 0.14 (0.45)	− 0.06 (0.74)	0.34 (0.06)

Italicized values indicate *p* < 0.05. Correlations analyses were only done for the 800-m measure because of the large number of 0 counts for schools at 400 m

^*^Adjusted for commercial land area using partial correlation

## Discussion

Prior to the January 1, 2020 regulatory change, the majority (83.3%) of secondary schools in London, Ontario, had a retailer within 800 m that advertised vaping products. Cole et al. (2019) found in their study of four Canadian provinces (Ontario, Quebec, Alberta, and British Columbia) that the mean number of vape shops in 2017 was fewer than one, even at 1500 m from schools. In medium-sized urban cities in Ontario, like London, they found that 33% of schools had a vape shop within 1500 m and only 20% had one within 1000 m. However, as previously noted, Cole et al. (2019) did not include convenience stores and gas stations. Our data suggest that, as vaping products containing nicotine became more widely available at various retail locations, the number of retailers selling, and therefore advertising, these products increased in the immediate areas surrounding schools. This is concerning as research suggests that changes in the environments surrounding schools can modify the potential for youth exposure to products that may be harmful (Wilson et al. 2006). This may impact subjective experiences of space and place and thus impact health behaviours.

A significant and large reduction in vaping advertisements surrounding secondary schools followed the January 1, 2020 policy change. This represents a reduced potential for youth to be exposed to vaping advertisements in the areas near their schools. It also supports similar initiatives in other provinces and jurisdictions. Two such examples are federal regulations introduced July 2020 that prohibit the promotion of vaping products that may be seen or heard by young people (e.g., at recreational facilities and transit shelters) (Government of Canada 2020b) and a further amendment to the *Smoke-Free Ontario Act* (July 2020) that prohibits specialty vape shops from having indoor displays and promotions that are visible from the outside (Government of Ontario 2020). Reductions in exposure to vaping advertisements in retail settings may impact on the perceived norms, perceived ease of access, and vaping behaviours of youth (Giovenco et al. 2016a, b). Although this study found compliance to new regulations was high among convenience stores and gas stations, continued monitoring of retailers is important to ensure sustained compliance. Future research should monitor for potential shifts in manufacturer marketing behaviours that may occur in response to changing policies and regulations.

Prior to the advertising ban, a positive correlation was found between vaping advertisement measures and school-level residential instability, when adjusting for commercial land use. These significant relationships may result in disproportionate health impacts due to greater exposure to vaping advertisements (Venugopal et al. 2020). On the other hand, following the advertising ban, the relationship between vaping advertisement measures and residential instability was no longer significant, suggesting that the ban reduced socio-demographic inequities in potential vaping advertisement exposure. Prior research suggests that higher socio-economic status communities may be more desirable for vape retailers as residents are more likely to adopt newer technologies, and have a greater disposable income (Simon et al. 2018). In the context of secondary schools in London, Ontario, however, our research did not provide evidence to support this suggestion. It is nevertheless important to note that we examined overall vaping advertisements and did not focus solely on specialty shops. Specialty shops may differ from convenience stores and gas stations (which may also sell tobacco products) in terms of the socio-demographic characteristics of areas in which they locate and products they promote. Our findings suggest that regulations on marketing have the potential to reduce inequities in youth vaping advertisement exposure in the environments surrounding schools.

There are several limitations to this study. First, the audits only examined store exteriors, but store interiors also represent a potential source of vaping advertisement exposure for youth. Second, while London, Ontario, is often described as “the most average place in all of Canada” (D’Ascanio 2019), study results are not generalizable across all of Ontario. Moreover, we did not analyze the specific content of the advertisements. Future research is needed to further explore the content of vaping advertisements and what products are being advertised where. For example, some promotions may be geared towards non-smoking youth, while others may focus on tobacco smoking cessation. Additionally, with cannabis legal in Canada, some advertisements may promote the use of cannabis with a vaping device; this warrants further research. It is also important to note that we examined associations between vaping advertisements surrounding schools and *school* (i.e., student body) socio-demographic characteristics. Future studies are needed that examine relationships between potential exposure to vaping advertisements and residential neighbourhood socio-demographic characteristics. While the environment surrounding schools is an influential venue for advertising exposure, residential environments and journeys to and from school can render equally important and fundamentally different exposure outcomes. Finally, although our findings illustrate that the potential for vaping advertisement exposure in the areas surrounding secondary schools was reduced after a marketing policy change, future studies are needed to examine whether such changes influence youth vaping behaviours.

Despite these limitations, our study has several strengths. It is the first study to examine the impact of a retail vaping advertisement ban on potential advertising exposure in the areas surrounding schools. Additionally, we included both specialty shops as well as convenience stores and gas stations. The omission of the latter is a noted limitation of previous Canadian studies that examine the geographies of vape retailers surrounding schools (Cole et al. 2019; Robitaille et al. 2019). Third, in addition to online searches, we used a team approach with two researchers working together to complete street audits of all commercial areas, to ensure no retailers were missed.

## Conclusion

We found that the January 1, 2020 provincial ban of vaping advertisements at retailers, apart from specialty shops, significantly reduced vaping promotions in the areas surrounding secondary schools in London, Ontario. We also found evidence that the ban reduced socio-demographic inequalities in potential youth exposure to marketing of vape products in a mid-sized city, thus advancing health equity. Future research should assess whether other Canadian provincial jurisdictions that adopted similar restrictions were able to reduce potential exposures of vaping advertisements among youth. Given the rapidly changing regulatory landscape of vaping in Canada, monitoring of the geographic accessibility and promotion of vaping products is warranted, especially given the complex interplay among federal, provincial, and municipal spheres of responsibility over the issue.

## References

[CR1] Azagba S, Manzione L (2020). Retail outlets and point-of-sale marketing of alternative tobacco products: another threat to tobacco control. The Journal of Adolescent Health.

[CR2] Boak A, Elton-Marshall T, Mann RE, Hamilton HA (2020). Drug use among Ontario students, 1977–2019: detailed findings from the Ontario Student Drug Use and Health Survey (OSDUHS).

[CR3] Bowman DD, Minaker LM, Simpson BJ, Gilliland JA (2019). Development of a teen-informed coding tool to measure the power of food advertisements. International Journal of Environmental Research and Public Health.

[CR4] Cho YJ, Thrasher JF, Reid JL, Hitchman S, Hammond D (2019). Youth self-reported exposure to and perceptions of vaping advertisements: findings from the 2017 International Tobacco Control Youth Tobacco and Vaping Survey. Preventive Medicine.

[CR5] Cohen J (1988). Statistical power analysis for the behavioral sciences.

[CR6] Cole AG, Aleyan S, Leatherdale ST (2019). Exploring the association between E-cigarette retailer proximity and density to schools and youth E-cigarette use. Preventive Medical Reports.

[CR7] Cole, A. G., Aleyan, S., Battista, K., & Leatherdale, S. T. (2020). Trends in youth e-cigarette and cigarette use between 2013 and 2019: insights from repeat cross-sectional data from the COMPASS study. *Canadian Journal of Public Health*. 10.17269/s41997-020-00389-0. 10.17269/s41997-020-00389-0 PMC7851234 32804379

[CR8] D’Ascanio N. (2019). This city is the most average place in all of Canada. Readers Digest Canada. https://www.readersdigest.ca/travel/canada/most-average-place-in-canada/.

[CR9] Dai H, Hao J (2017). Geographic density and proximity of vape shops to colleges in the USA. Tobacco Control.

[CR10] Field AP, Miles J, Field Z (2012). Discovering statistics using R.

[CR11] Fradette K, Keselman HJ, Lix L, Algina J, Wilcox RR (2003). Conventional and robust paired and independent-samples t tests: type I error and power rates. Journal of Modern Applied Statistical Methods.

[CR12] Giovenco DP, Casseus M, Duncan DT, Coups EJ, Lewis MJ, Delnevo CD (2016). Association between electronic cigarette marketing near schools and e-cigarette use among youth. The Journal of Adolescent Health.

[CR13] Giovenco DP, Duncan DT, Coups EJ, Lewis MJ, Delnevo CD (2016). Census tract correlates of vape shop locations in New Jersey. Health & Place.

[CR14] Government of Canada. (2020a). *Vaping product regulations*. https://www.canada.ca/en/health-canada/services/smoking-tobacco/vaping/product-safety-regulation.html.

[CR15] Government of Canada. (2020b). *Final Vaping Products Promotion Regulations (VPPR)*. https://www.canada.ca/en/health-canada/news/2020/07/final-vaping-products-promotion-regulations-vppr.html.

[CR16] Government of Ontario. (2020). *Ontario Protecting Children and Youth from Vaping* https://news.ontario.ca/en/backgrounder/55979/ontario-protecting-children-and-youth-from-vaping.

[CR17] Hammond D, White CM, Czoli CD, Martin CL, Magennis P, Shiplo S (2015). Retail availability and marketing of electronic cigarettes in Canada. Canadian Journal of Public Health.

[CR18] Kim S (2015). ppcor: an R package for a fast calculation to semi-partial correlation coefficients. Communications for Statistical Applications and Methods.

[CR19] Krugman DM (2016). Understanding the impact that marketing, advertising, and promotion have on adolescent e-cigarette behavior. The Journal of Adolescent Health.

[CR20] Mantey DS, Cooper MR, Clendennen SL, Pasch KE, Perry CL (2016). E-cigarette marketing exposure is associated with e-cigarette use among US youth. The Journal of Adolescent Health.

[CR21] Martin G, Inchley J, Marshall A, Shortt N, Currie C (2019). The neighbourhood social environment and alcohol use among urban and rural Scottish adolescents. International Journal of Public Health.

[CR22] Matheson FI, Dunn JR, Smith KL, Moineddin R, Glazier RH (2012). Development of the Canadian Marginalization Index: a new tool for the study of inequality. Canadian Journal of Public Health.

[CR23] Miyashita, L., & Foley, G. (2020). E-cigarettes and respiratory health: the latest evidence. *The Journal of Physiology*. 10.1113/JP279526 32495367

[CR24] National Academies of Sciences and Engineering (2018). Public health consequences of e-cigarettes.

[CR25] Ontario Ministry of Health. (2019). *Protecting youth from the dangers of vaping: Ontario banning the promotion of vaping products outside of specialty stores [News Release].* https://news.ontario.ca/mohltc/en/2019/10/protecting-youth-from-the-dangers-of-vaping.html.

[CR26] Robitaille É, Bergeron P, Houde M (2019). Analysis of the geographical accessibility of vape shops in the vicinity of Quebec’s secondary and college educational institutions. Health Promotion and Chronic Disease Prevention in Canada: Research, Policy and Practice.

[CR27] Simon P, Camenga DR, Morean ME, Kong G, Bold KW, Cavallo DA, Krishnan-Sarin S (2018). Socioeconomic status and adolescent e-cigarette use: the mediating role of e-cigarette advertisement exposure. Preventive Medicine.

[CR28] Statistics Canada. (2019). The Canadian index of multiple deprivation. Statistics Canada catalogue no. 45-20-0001*.*

[CR29] Statistics Canada. (2020). Canadian Tobacco and Nicotine Survey, 2019. https://www150.statcan.gc.ca/n1/daily-quotidien/200305/dq200305a-eng.htm.

[CR30] Venugopal PD, Morse AL, Tworek C, Chang HW (2020). Socioeconomic disparities in vape shop density and proximity to public schools in the conterminous United States, 2018. Health Promotion Practice.

[CR31] Wilson DH, Gilliland J, Ross NA, Derevensky J, Gupta R (2006). Video lottery terminal access and gambling among high school students in Montreal. Canadian Journal of Public Health.

